# Estimation of Factors Affecting Burnout in Greek Dentists before and during the COVID-19 Pandemic

**DOI:** 10.3390/dj10060108

**Published:** 2022-06-13

**Authors:** Maria Antoniadou

**Affiliations:** Dental School, National and Kapodistrian University of Athens, GR-115 27 Athens, Greece; mantonia@dent.uoa.gr; Tel.: +30-6944342546

**Keywords:** burnout syndrome, COVID-19, dental professionals, occupational dentistry, pandemic management, stress

## Abstract

This study is a comprehensive, cross-sectional survey in occupational burnout, career satisfaction, and quality of life conducted in March 2021 among dentists in the vast area of metropolitan Athens, Greece. Data were collected using a self-reported questionnaire based on the Copenhagen Questionnaire (CQ) for assessing work stress and the Maslach Burnout Inventory-Human Service Survey (MBI-HSS) for evaluating occupational burnout. Using the independent *t*-test, ANOVA, Pearson’s correlation, and multiple linear regression, 804 valid questionnaires were analyzed. During the pandemic, personal exhaustion was affected by gender (*b* = 1.862, *p* = 0.0001), age (*b* = −0.598, *p* = 0.0001), number of children (*b* = −0.886, *p* = 0.020) and higher degree (*b* = −0.450, *p* = 0.012). Exhaustion due to working with patients was affected by gender *(b* = 0.662 *p* = 0.0001), age (*b* = −0.513, *p* = 0.0001), number of children (*b* = −0.701, *p* = 0.0001), higher degree (*b* = −0.207, *p* = 0.028) and years in practice (*b* = 0.408 *p* = 0.0001). Males were more prone to unhappiness, dissatisfaction, and professional physical and emotional exhaustion, but personal resources through higher education, beliefs, values, and hobbies can offer a preventive shield to all dental professionals. Economic management issues can also enhance dentists’ satisfaction and feeling of safety in a rapidly changing environment.

## 1. Introduction

Burnout is described as the result of exhaustion, disappointment, and loss of interest in work, especially in service delivery where direct contact with other people is involved [1], as in the oral health care environment. This multifactorial syndrome makes dentists lose their will and energy to work. Emotional distress is the initial symptom, while physical and mental exhaustion follows [2]. Health problems related to stress and burnout among dentists and high levels of job dissatisfaction, depression, low quality of life, and moderate prevalence of burnout in several parts of the world have been reported before the COVID-19 pandemic emerged [3,4]. It has been well documented so far that the dental work process is characterized by aspects likely to influence job satisfaction and the well-being of dentists, such as productivity, stress level, salary, other economic issues, resource, or equipment shortage, extensive working hours, lack of free personal time and perceived staff quality [3]. Other factors influencing burnout in the dental field are younger age, male gender, student status, high job strain/working hours, those enrolled in clinical degree programs, and certain personality types [5]. Thus, dentists are expected to be constantly exposed to occupational stress with a high risk of burnout, which is strongly associated with job dissatisfaction, depression, rejection [6,7], and early retirement [8].

COVID-19 pandemic changed the dental routine of the private and public oral health sector. Revised safety protocols and obligatory air and water quality restrictions changed dental offices’ time management, strategic management, and clinical procedures [9,10,11,12]. Dentists’ daily routine was overloaded with protective equipment that brought extra working hours and economic needs, resulting in a “working more to cover less” situation that could absorb energy and cause extra stress [13,14]. Most relevant studies report a lack of dentists’ preparedness to confront a highly infectious respiratory disease [13,15]. The new level of protective armamentarium and updated operational guidance and policies required necessitated the delivery of new knowledge, skills, and equipment that put under question the sustainability of their practices and necessitated a robust strategic plan [15,16,17]. Although employees in public oral health sectors appear to be less prone to burnout [14,16], dentists in the private dental sector were facing, at the same time, more responsibilities, demanding economic reinforcement of the business while patients were reluctant or unable to spend [12,18,19,20]. This has led, in some cases, to selling practices or, in an extreme sense, ultimately leaving the profession [13,21]. For those dentists still working, the direct negative effect of burnout is the reduced quality of dental services. Even before the pandemic, it was reported that the absence of personal time, the relationships with dental patients, and the workload in the dental office were important factors of personal judgment with the risk of practicing dental procedures unsafely [22,23], and was a primary source of malpractice and low quality in dental services [4,24]. This was aggravated during the pandemic, where it was reported that dentists with a high burnout risk were more likely to report concern over a perceived error within the last six months [25].

The prevalence of burnout in dentists was another issue thoroughly investigated during the pandemic and found significant discrepancies. Dentists’ anxiety-related symptoms were, in general, aggravated, reaching in some cases more than 70% [14], their depression-related symptoms had overcome 60% [14], and stress was over 80% [14,26,27]. It was reported that only 27% of dentists announced significant depressive symptomatology [28], which makes us wonder about different factors influencing locally, ethnically, or culturing the phenomenon. For example, social factors such as being in a committed relationship and having higher scores for self-efficacy can lead to lower psychological distress [29], but the economic status of dentists [18], lack of staff [19], or perfectionism issues [30], could import different findings in burnout levels.

Even though there is documentation for pandemic-caused burnout discrepancies almost all over the world [31,32,33,34,35], the relevant impact in a sample of Greek dentists was investigated, since different factors seem to be related to the severity of the phenomenon in different countries. As relevant data show, Greece was one of the countries least affected by the COVID-19 pandemic worldwide in 2020 [36]. This fact could influence the way dentists face the pandemic and the fears generated from it. Thus, the present study aims to investigate burnout status and factors affecting it during the second pandemic lockdown (March–April 2021) in a sample of Greek dentists. More specifically, it aimed to assess the potential effect of different demographic variables, such as social and economic status, level of education, and other risk factors, as well as ethical limitations on personal and emotional exhaustion while working with patients during the second lockdown after the coronavirus outbreak, in March 2021. A secondary goal is to raise awareness of the risk of burnout in this population group.

## 2. Materials and Methods

### 2.1. Study Methodology Background

For this study, all relevant literature on the three components of burnout was reviewed: emotional exhaustion (EE), characterized as emotional tension leading to a feeling of exhaustion; depersonalization (DP), i.e., a hostile, often indifferent, or cynic attitude toward clients; and low personal accomplishment (PA), which interferes with the interpersonal skills required in the dental professional practice [2]. It was apparent that while physical exhaustion might be a sign of burnout, emotional exhaustion is the core aspect of this condition [37] that had to be investigated in this study.

There are specific questionnaires used in the relevant literature for assessing burnout. Regarding burnout of dentists, the 22-point questionnaire of Maslach (Μaslach Burnout Inventory-MBI) is usually used [38]. The Maslach burnout inventory in human services (MBI-HSS) [38] is considered the most established tool for measuring burnout in dentistry and other fields where human services are involved (MBI-HSS) [39,40,41]. MBI-HSS is a tool suitable for assessing the dimensions of the EE, at least among health facility staff. Currently, it is used systematically during the COVID-19 pandemic as the most helpful tool for recording the phenomenon in dentists [41]. At the same time, though, the MBI-HSS has been extensively criticized by researchers for various reasons, such as an unclear relationship between the tool and the concept of burnout, since it uses questions mainly related to American culture that would probably not be generalized in the same sense into other populations [42]. Kristensen et al. [42], developed for this reason “The Copenhagen Burnout Questionnaire (CBI)” which measures three factors: personal burnout, exhaustion of the provider, and exhaustion from the influence of patients/customers with satisfactory reliability and validity for all subscales [42]. It is reported that the CBI tool is most often used to measure burnout in staff working in complex medical settings [43].

### 2.2. Questionnaire Used in this Study

For this investigation, a comprehensive questionnaire was used for data gathering, based on the following questionnaires: (1) the Maslach questionnaire referring to burnout, compassion satisfaction, and level of secondary traumatic stress, (2) the CBI questionnaire referring to coping strategies (task-focused, emotion-focused, or avoidance-focused coping). Specific demographic and professional variables were also included. The questionnaire consisted of three main sections: The first section included items that explored primary demographic data of the dentists such as gender, age, years in the profession, a higher degree in the field of dentistry, the existence of a second academic degree, marital and economic status, number of children, exercising of a dental specialization and mode of working relationship with the practice localization (employee vs. business owner). The second part consisted of questions referring to dentists’ physical and emotional status before the pandemic. The third part, as with the previous, was based on questions from surveys (1) and (2) and addressed issues during the pandemic and the impact that the pandemic had on dentists.

The questionnaire was specifically developed for the study. Translation of the original questionnaires used in the study was carried out by one Greek–English and one English–Greek authenticated translator. Content validity was confirmed by a panel of experts (4 university professors), all target population members. During multiple consultations with them, we elicited the relevant issues, drafted the questions from the above questionnaires, adjusted them based on their comments, and piloted a final draft version to assure the clarity and appropriateness of the questionnaire. Prior to the start of the data collection, the survey was pilot tested on 20 dentists to assess the comprehensibility and usability of all included sections. None of them reported any difficulties in answering included items, and they found each component to be relevant to the current situation, with the average time for survey completion 15–20 min. The final survey version was then uploaded to a Google forms page, and the link was sent with all relevant information of completion and consent forms to the secretariat of the dental associations of Athens and Piraeus to forward to members.

Question examples: Dentists who marked “strongly agree” or “agree” in response to the survey items “Would you like any of your children to follow your profession? (Even if you don’t have children now)”, “If you were asked today, would you choose the dental profession?”, “I believe that I can make a difference for the improvement of society through the dental profession” were satisfied with their career.

Those reported “strongly agree” or “agree” in response to the survey items “I was happy before the pandemic”, I think I do not work long hours, and I have a personal life”, “ I am pleased because I feel able to evolve through my profession as a person and as a scientist”, “I am the person I have always wanted to be”, were considered satisfied with the work–life integration, respectively.

### 2.3. Sample

All dentists working in metropolitan Athens, the capital of Greece (the area included the city of Athens and Piraeus Port) (*n* = 6300), were asked to participate in an online questionnaire from 20–30 of April 2021. The questionnaire was approved by the scientific board of the relevant dental associations, which gave approval via email (protocol number 704, 17 April 2021). The secretariat has emailed the invitation twice with the link of the Google forms questionnaire to all members so that every member had an equal opportunity to participate. At the same time, protection of their privacy was also guaranteed.

### 2.4. Statistical Analysis

The outcome variables were the percent frequencies and the mean scores for each survey question or summary scores of questions with similar content. The data were normally distributed according to the Shapiro–Wilk test for normality (*p* > 0.05). Two types of inferential analysis were conducted; first, we assessed the potential differences in questions’ frequencies before and during the pandemic using Cochran’s Q test. In addition, multiple linear regression models were applied to have as predictors the demographic characteristics of the sample and survey questions of interest, and as outcome variables, the survey questions mean scores. All reported probability values (*p* values) were compared with a significance level of 5% (*p* < 0.05). The analysis of coded data was carried out using the IBM SPSS Statistics for Windows, Version 27.0. (IBM Corp., Armonk, NY, USA).

## 3. Results

We received 804 valid responses (response rate = 12.76%). The internal consistency of the survey was very satisfactory (Cronbach’s alpha: 0.81). Regarding the sample, 44.92% were males (N_1_ = 361), 55% women (N_2_ = 442) and 0.1% other (N_3_ = 1). The age categories consisted of participants that were: (a) 9.2% up to 30 years of age (N_4_ = 74), (b) 17.2% from 31–40 years of age (N_5_ = 138), (c) 32.7% from 41–50 (N_6_ = 263), (d) 29% from 51–60 (N_7_ = 233), (e) 11.9% 60 years and above (N_8_ = 96). In addition, 64,8% of the participants were married (N_9_ = 521), 23.5% unmarried (N_10_ = 189), 8.7% divorced (N_11_ = 70) and other sorts of personal relationships 3% (N_12_ = 24); 30.7% of the participants (N_13_ = 247) had no children, while 60.3% had 1–2 children (N_14_ = 485) and 9% had 3 and more children (N_15_ = 72). Furthermore, 62.9% of the participants had basic dental education (N_16_ = 506), 28.4% had a master’s degree (N_17_ = 228) and 8.7% had a PhD degree in dentistry (N_18_ = 70). In addition, 17.2% of the participants had a second degree in another science (N_19_ = 138) while 82.8% had qualifications only in dentistry (N_20_ = 666).

Furthermore, 75.9% of the participants exercised general dentistry (N_21_ = 610), 3.2% esthetic dentistry (N_22_ = 26), 1.1% oral surgery (N_23_ = 9), 4.1% endodontics (N_24_ = 33), 3.9% orthodontics (N_25_ = 31), 4.2% pedodontics (N_26_ = 34), 2.6% periodontology (N_27_ = 21), 3.6% prosthetics (N_28_ = 29), 0.5% stomatology (N_29_ = 4), 0.9% something else (N_30_ = 7). Regarding years in the profession, 20.1% had exercised dentistry for 1–10 years (N_31_ = 162), 30.3% for 11–20 years (N_32_ = 244), 27.2% for 21–30 years (N_33_ = 219) and 22.3% for 31 and above years (N_34_ = 179). Concerning the economic status of the participants 20.3% earned under EUR 15000 per year (N35 = 163), 27.1% earned EUR 15001–25000 (N_36_ = 218), 32.7% between EUR 25001–50000 (N_37_ = 263), and 15.5% above EUR 50000 (N_38_ = 125).

Most of them had a private dental office (78.7%, N_40_ = 633) while 3.9% were working in public hospitals (N_41_ = 31), 1.7% were academics (N_42_ = 14), 3.5% were also offering services outside their office as freelancers (N_43_ = 28), 3.4% were only freelancers without a private office of their own (N_44_ = 27), 4.2% were working at the same office with another dentist (N_45_= 34), 3.7% were employees in somebody else’s office (N_46_ = 30) and 0.9% were differently occupied (N_47_ = 7). Of those that had their private office, 56.3% had no employee (N_48_ = 453), 21.1% had one employee (N_49_ = 170), and 8.1% had 2–3 employees N_50_ = 65). Four employees and above were reported by 3% (N_51_ = 24) while no office was declared by 91% of them (N_52_ = 91).

In Table 1 all significant differences before and during pandemic frequencies are depicted (Cochran’s Q test, *p* < 0.05). More specifically, participants reported that they were feeling tired to a meager degree before (15.8%) compared to during the pandemic (9.6%), while they were feeling tired to a very high degree 4.9% before, compared to 26.5% during the pandemic (almost 5.5 times up). They were also feeling emotionally exhausted at a significant level before the pandemic; 27.7% reported being emotionally exhausted to a meager degree before and almost half the percentage during the pandemic (10.2%). On the contrary, 10.8% declared a high degree of emotional exhaustion before and 31% after, and 3% a very high degree before with 25.5% after (8.5 times up during the pandemic). Although not statistically significant, it is interesting to mention that, concerning having enough energy for family and friends during leisure time, 5.8% reported a very low degree before, which was almost tripled during the pandemic (14.7%), and all levels of responses were diminishing in intensity during the pandemic, suggesting that the energy flow was significantly diminishing for all participants due to this cause.

Percentages before and during the pandemic were also statistically significant for exhaustion due to working with patients. “Finding it hard to work with patients” was reported to a high/very high degree before by the 5.5% of the sample, and this increased to 21.8% during the pandemic (5 times up). Almost half of them (55.8%) did not have problems working with patients before the pandemic (to a very low degree), but this percentage seems to diminish during the pandemic (33.3%), with more dentists suggesting finding it hard to work with patients in all levels of answering.

Differences in dentists reporting being happy before than during the pandemic were statistically significant too. Those reporting being happy to a very high degree during the pandemic was half the percentage (8.3%) than before the pandemic (17%), while being happy to a high degree before was 30.7% than 18.8% during the pandemic. Thus, perceived happiness was also diminishing during the pandemic. At the same time, participants “feeling trapped by the work in the dental office” to a very high degree were almost doubled in a statistically significant level (15.2% during than 6.2% before) and to a high degree 21% during than 16.4% before.

As far as it concerns answers to the item “being satisfied by remuneration”, those reporting a very low degree before were 9%, and this percentage has almost doubled during the pandemic (18.3%). In addition, 19.9% reported remuneration satisfaction to a low degree before and 24.5% during the pandemic. In all levels of answers, the satisfaction from remuneration was significantly diminishing during the pandemic.

Answering that “being anxious when waking up about not catching up with everything” remained almost at the same levels before and during the pandemic, except for the number of highly anxious dentists who almost doubled during the pandemic (5% before vs. 9.5% during the pandemic).

Regression analysis results/models about factors affecting personal exhaustion are shown in Table 2 and Table 3. More specifically, in Table 2, personal exhaustion results are shown. Gender was a predictor of physical and emotional exhaustion before the pandemic; Males were more prone to physical tiredness than females (*b* = 0.552, *p* = 0.0001), while years in the profession affected the sample population positively, meaning that more years in the profession affected these issues (*b* = −0.202, *p* = 0.014). In addition, filling tired and emotionally exhausted was more possible during the pandemic than before (*b* = 0.463, *p* = 0.0001). Furthermore, the combined summary score of “feeling tired”, & “feeling emotionally exhausted”, & “feeling dry or puffed at the end of the day”, & “being on the verge of illness” was found to increase during the pandemic in males (*b* = 1.862, *p* = 0.0001) but diminished by age (*b* = −0.598, *p* = 0.0001), number of children (*b* = −0.886, *p* = 0.020) and the existence of a higher degree (*b* = −0.450, *p* = 0.012).

“Being annoyed by the work in the dental office” regardless of the pandemic period was affected negatively by the number of children (*b* = −0.277, *p* = 0.004), meaning the more children, the less annoyed a dentist was in the office.

“Having enough energy for family and friends during leisure time” before the pandemic was affected by gender; males were less prone to have enough energy (*b* = −0.196, *p* = 0.015) before the pandemic than females. Years in the profession were positively affecting the level of energy (*b* = 0.143, *p* = 0.003), while the number of children (*b* = −0.048, *p* = 0.025) and the additional higher degree affected negatively (*b* = −0.037, *p* = 0.029). During the pandemic, “having not enough energy for family and friends during leisure time” was affected by gender, where males are expected to be affected more than females (*b* = 0.435, *p* = 0.0001). In addition, during the pandemic, age was a negative factor (*b* = −0.093, *p* = 0.036), meaning that the older the dentist, the less energy they were expected to have. Further, the number of children negatively affected the energy (*b* = −0.360, *p* = 0.001), meaning that the more children, the less energy the dentists had. The exact impact of the existence of a higher degree (*b* = −0.179, *p* = 0.001) and a postgraduate degree (additional to dentistry) (*b* = −0.258, *p* = 0.034) suggested that for those having studied more, less energy is expected for family and friends during the pandemic.

In Table 3, data on exhaustion because of working with patients are shown. A summary score of questions concerning “finding it difficult to deal with patients” & “wondering at times how much longer I can continue to work with patients” during the pandemic were associated with the summary score of the following questions before the pandemic: “I found it difficult to deal with patients” & “I feel that working with patients reduces my energy regardless of the period of the pandemic we are experiencing” & “I believe that I give more than I get when I work with patients regardless of the period of the pandemic we are experiencing” and “I am tired of working with patients regardless of the period of the pandemic we are experiencing”. Dentists who answered the second group of questions positively were more prone to be affected and answer positively to the first group of questions (*b* = 0.629, *p* = 0.0001).

The summary score of questions such as “before the pandemic, I found it difficult to deal with patients”, & “I feel that working with patients reduces my energy regardless of the period of the pandemic we are experiencing”, & “I believe that I give more than I get when I work with patients regardless of the period of the pandemic we are experiencing” & “I am tired of working with patients regardless of the pandemic period” were also negatively impacted by age (*b* = −0.390, *p* = 0.003) and number of children (*b* = −0.822, *p* = 0.012). Τhis means that the more children a dentist has and the greater their age is, the less likely they are to answer the previous questions positively.

During the pandemic, answers to questions such as “finding it difficult to deal with patients” and “wondering at times how much longer I can continue to work with patients” were affected positively by gender (*b* = 0.662, *p* = 0.0001); thus, males are more prone to answer positively to these questions, while age (*b* = −0.513, *p* = 0.0001), the number of children (*b* = −0.701, *p* = 0.0001) and higher degree (*b* = −0.207, *p* = 0.028) negatively affected these questions. On the contrary, the years of practice were affected positively (*b* = 0.408, *p* = 0.0001).

## 4. Discussion

The results of our study indicate that physical and emotional exhaustion in our sample were at a very high level and were 5.5 and 8.5 times up respectively during the pandemic than before. Greek dentists seemed to suffer from exhaustion due to COVID-19, with high EE being the most common finding, while other high dimensions of burnout-DP and PA additionally occurred frequently as is also discussed in numerous relevant studies across the globe, before [5,7] and during COVID-19 pandemic [14,28,44,45,46,47]. However, this phenomenon was reported in our study at a lower level than elsewhere [34,35,48,49].

Several demographic variables (gender, age, educational level, number of children, and marital status) and job-related variables (work practice experience duration, specialist status, numbers of employees, income) were identified as being associated with some aspects of the burnout syndrome in our study. It has been already reported that gender differences reflect different working conditions for men and women [50]. Women are more attached to their families and are often expected to work part-time or even stop working as a dentist while raising their children [22,51,52]. Some authors report that female gender predicts more emotional exhaustion [23,53,54,55] while a few studies describe no statistically significant gender differences before the pandemic [56,57,58]. In our study males were more prone to physical and personal exhaustion and had less energy for family and friends before and during the pandemic as discussed also elsewhere [39]. This could be attributed to the possibility of working more hours during the pandemic while female professionals have probably remained at home taking care of children [10,12,14,40]. During the pandemic, physical and emotional exhaustion as well as exhaustion due to working with patients have also increased significantly in men as they felt somehow trapped due to difficulties and insecurities of the period while they were simultaneously obliged to support their family economically [59].

The educational level in dentistry was another critical predictor of these issues. It is possible that dentists with postgraduate qualifications could be involved in other pursuits at work that have been shown to protect against burnout, such as teaching, research, and administrative duties, which are built into their working day, giving greater variety. Interestingly, a higher educational level was not predictive of lower burnout in any of the domains in the regression analysis of the data obtained by Croucher et al. [60]. Contrastingly, possession of a postgraduate qualification was significantly associated with overall work engagement, indicating that this resource possibly has a buffering effect against the development of burnout [56]. Before the pandemic, it was mentioned that dentists with postgraduate qualifications had significant lower burnout scores than the graduates [61], something that was also mentioned by Maslach, who reported that burnout is found for providers who have completed college but have not had any postgraduate training [62]. Our data also prove that acquiring a postgraduate degree in dentistry is a protective factor during stressful situations, giving professionals greater enumeration and consequently let them address less depressed moods. [63].

The influence of age as a predictor of burnout has been also reported before. Maslach reasoned that people are more stable and mature with increased age and have a more balanced perspective on life [64]. Usually, dentists under the age of 30 years practice dentistry for up to four years [62,65] and are trying to find their business steps in the oral healthcare field, including organizing their practice economically. Therefore, the relevant responsibilities make the age group (25–35 years) more vulnerable to burnout [39,55,57,66] as also found here. Information and economic struggle overload because of the closure of their practices for several months could be the reason [15]. Although discrepancies in the age group affected more by burnout before the pandemic are discussed elsewhere [22,67,68], in our sample older dentists seem to have internal sources to help them surpass economic and fearful situations (values, beliefs etc.).

Years of practice, corresponding to work experience, may act as a direct determinant or a moderator in the burnout process. Only a few studies have shown that work experience is associated with high levels of burnout [55,69]. In other cases, practitioners working 16 to 22 years showed increased emotional exhaustion (EE), but also higher levels of personal accomplishment (PA) [52], which compensated for emotional tiredness. This was in line with a study among academicians where the professors had the highest PA scores [57]. Contradicting findings have been reported so far on the issue that less experience in the profession seems to enforce personal and emotional exhaustion [66]. Elsewhere, young dentists with short work histories and older dentists with long professional experiences experienced burnout symptoms to the same extent [70]. Work experience only guarantees ways of keeping their energy but does not help them feel less exhausted when working with patients [71]. In the relevant studies, usually, younger dentists (up to 40 years) have a significantly higher depersonalization mean score (DP) than older ones (40 years and older) [39,72], but a significant difference between age groups was not observed in our study. Our findings confirm the results of earlier studies, which showed that burnout most often occurs in the first years of one’s professional career [5,20,72,73,74]. Accordingly, dentists in the older age range of our sample could be those who had survived the early professional stress and, at present, they do well in their professional activity, as also mentioned elsewhere [12,40,72]. These findings coexist with variation in the EE dimension by years of work experience, which was revealed in previous studies [72,73], regardless of the contrary data [75]. Interesting enough, higher educational level (having a second university degree) also seems to be a protective factor against exhaustion, even though it may drain the physical energy, maybe because well-educated dentists find more inner worth and self-compliance than others who feel a lower level of self-achievement [76].

The number of children seems also to be a predictor of exhaustion for dentists in our study. Having more children corresponded with low energy before and during pandemic but is a protective factor for emotional exhaustion and exhaustion due to working with patients as it seems to enhance emotional intelligence and personal resilience [62,77]. This is consistent with data suggesting marriage and having children as protective factors against burnout [78].

There was also variation in the burnout level by marital status in our study. It is also mentioned elsewhere that newly married dentists were more likely to rate poorer personal accomplishment. In the literature, several authors [22,67,79], suggested that married people would be more resistant to burnout because they exercise emotional intelligence and show remarkable ability to face emotional problems [29,80]. Other authors [81] identified no relationship between burnout manifestation and marital status. It is interesting, though, to report that happiness during the pandemic was inversely affected by marital status, suggesting that other factors can be more critical in determining the phenomenon in Greece.

Income was much less an essential predictor in our study, explained mainly by loss of earnings due to subsequent lockdowns. Our data show that negative beliefs or inability to receive the correct payment were a significant source of anxiety and disappointment in the profession, as also mentioned elsewhere [20,26]. Newly addressed protocols during the pandemic required extreme caution and preparedness by dental professionals [17,28] and costed more. This new reality augmented daily stress for most dentists worldwide [11] and in Greece [17,18]. Inevitably, the cost of personal protective equipment and the lower volume of patients due to subsequent closures posed a continued threat to business [18,21,82] with serious cash-flow problems or even bankruptcy issues. This was also the case for many small businesses worldwide [83].

Findings showed that the positive association between emotional overload and psychological distress was different among countries, suggesting a higher rate of intensity in Italy [26] compared to the UK [21], Australia [11], Colombia [40], Germany [12], Poland [48], Slovakia [19], Israel [9] and Lithuania [72]. Greek dentists seem less exhausted, possibly since Greece was less affected by the pandemic until the study (March 2021). This finding is consistent with the suggestion that the interaction variable of the subjective overload and psychological distress was significantly associated with a particular country [13], presumably due to specific background issues such as social, cultural, and environmental factors, also discussed elsewhere [34].

Improving physicians’ wellness and implementing self-care strategies is a multifactorial process [84] and includes attention to both personal and professional self-care, while personal resilience alone will not suffice [13]. Personal self-care refers to strategies for individual physicians to better care for themselves. It starts with recognizing that people have multiple personal dimensions to which to attend to live a “good” life [85], including inner lives, families, work, community, and spirituality. Strategies for personal self-care include prioritizing close relationships such as those with family; [86,87], maintaining a healthy lifestyle by ensuring adequate sleep, regular exercise, and time for vacations; [88] fostering recreational activities and hobbies; [89,90] practicing mindfulness and meditation; [91,92], pursuing spiritual development [93] and relate to other professionals in supportive groups [94]. These experiences, if recognized and fostered, can, in turn, underscore the importance of self-care.

Finally, living in the present, cultivating meaningful personal and professional relationships, attending to spiritual life, and developing self-awareness might help in the direction of prevention over the phenomenon. Lastly, if truly embraced, the precepts and benefits of good self-care can be conveyed to the next generation of dentists, formally as such principles are integrated into training, and informally as preceptors, mentors or coaches model these attitudes and skills for their trainees/coachees. This is already proven to be an effective instrument for preparedness during periods of high uncertainty [28,95]. Only with such efforts can the increasingly challenging work of caring for dental patients in an unsafe working and economic environment become sustainable for those who choose to do it.

## 5. Limitations of the Study

Among the main limitations of the present study, its cross-sectional design does not allow for the direct identification of causal factors of job satisfaction or burnout. Furthermore, the pandemic is not over, so there is a possibility that dentists’ responses before the pandemic can be biased. Furthermore, since this is a self-administrated survey, there is always the risk of recall bias. It is also important to discuss the low response rate of our sample. According to data of the Hellenic Dental Association and the two dental associations that participated in the study, it seems that one out of seven dentists responded to our survey, which is a very satisfactory impact compared to relevant studies. Further, at the time the questionnaire was sent, dentists were preparing to reopen after a year’s closure and obviously they were quite busy and reluctant to spend time on the issue. Finally, the two dental associations that participated in the study have almost half of the dental professionals working currently in Greece, so they gather a very representative number of participants. So, the response rate of the sample, although rather low, could not affect the internal consistency of the survey if we also consider the Cronbach’s alpha index retrieved. Age distribution of the sum of dentists to whom the questionnaire was sent was followed in our sample, suggesting that all age groups were equally represented in our data. Nonetheless, the obtained information from our sample contributes to a better understanding of the phenomenon while it leaves the field open for more thorough studies with more complex designs.

## 6. Conclusions

In stressful situations such as a pandemic, occupational burnout can be influenced positively by age, educational level, marital status, and number of children. Males are more prone to unhappiness, dissatisfaction, physical and emotional exhaustion. Age, educational level and working experience affect the psychological status of dentists inhibiting burnout positively. Income was a significant predictor of burnout during the pandemic. Economic management issues can enhance dentists’ satisfaction and feeling of safety in a rapidly changing environment. A more thorough reflection on the working conditions of dentists in Greece is needed to improve the approach to causative factors.

## Figures and Tables

**Table 1:** Selected findings in burnout estimation before and during the pandemic.

Parameter	To a Very Low Degree		To a Low Degree		Somewhat		To a High Degree		To a Very High Degree	
	*n* (%)	*n* (%)	*n* (%)	*n* (%)	*n* (%)	*n* (%)	*n* (%)	*n* (%)	*n* (%)	*n* (%)
	Before	During	Before	During	Before	During	Before	During	Before	During
Feeling tired *	127 (15.8)	77 (9.6)	194 (24.1)	126 (15.7)	250 (31.1)	157 (19.5)	194 (24.1)	231 (28.7)	39 (4.9)	213 (26.5)
Feeling emotionally exhausted *	239 (27.7)	82 (10.2)	244 (30.3)	121 (15)	210 (26.1)	147 (18.3)	87 (10.8)	249 (31)	24 (3)	205 (25.5)
Having enough energy for family and friends during leisure time	47 (5.8)	118 (14.7)	117 (14.6)	163 (20.3)	225 (28)	164 (20.4)	259 (32.2)	217 (27)	156 (19.4)	142 (17.7)
Finding it hard to work with patients *	449 (55.8)	268 (33.3)	226 (28.1)	198 (24.6)	85 (10.6)	159 (19.8)	35 (4.4)	132 (16.4)	9 (1.1)	47 (5.8)
Being happy *	52 (6.5)	170 (21.1)	102 (12.7)	173 (21.5)	266 (33.1)	243 (30.2)	247 (30.7)	151 (18.8)	137 (17)	67 (8.3)
Feeling trapped by the work in the dental office *	228 (28.1)	188 (23.4)	205 (25.5)	169 (21)	191 (23.8)	156 (19.4)	132 (16.4)	169 (21)	50 (6.2)	122 (15.2)
Liking my job in the dental office *	24 (3)	63 (7.8)	49 (6.1)	96 (11.9)	125 (15.5)	176 (21.9)	288 (35.3)	267 (33.2)	318 (39.6)	202 (25.1)
Being satisfied with my professional performance	16 (2)	41 (5.1)	52 (6.5)	68 (8.5)	147 (18.3)	158 (19.7)	386 (48)	356 (44.3)	203 (25.2)	181 (22.5)
Being satisfied by my remuneration for the work I did and the responsibilities I took on *	72 (9)	147 (18.3)	160 (19.9)	199 (24.8)	280 (34.8)	226 (28.1)	220 (27.4)	170 (21.1)	72 (9)	62 (7.7)
Being anxious when I woke up that I wouldn’t catch up with everything	222 (27.6)	237 (29.5)	220 (27.4)	187 (23.3)	181 (22.5)	154 (19.2)	141 (17.50	150 (18.7)	40 (5)	76 (9.5)

* Significant differences between before and during the pandemic frequencies (Cochran’s Q test, *p* < 0.05).

**Table 2:** Personal exhaustion before and during pandemic (multiple linear regression modeling).

	Personal Exhaustion-Outcome Variables					
Predictors		**B**	**95% CI for B**		**P**	**Association**
	Summary score of “feeling tired before/feeling emotionally exhausted before the pandemic”					
	Gender	0.552	0.281	0.823	0.0001	Increasing in males
	Years in profession	−0.202	−0.364	−0.041	0.014	Negative (inverse)
	Summary score of “having enough energy for family and friends during leisure time before the pandemic					
	Gender	−0.196	−0.353	−0.038	0.015	Decreasing in males
	Years in profession	0.143	0.049	0.237	0.003	Positive (in tandem)
	Number of children	0.048	−0.141	0.237	0.025	Negative (inverse)
	Higher degree	0.037	−0.052	0.126	0.029	Negative (inverse)
	Summary score of “I am annoyed by my work in the dental office regardless of the period of the pandemic we are experiencing					
	Number of children	−0.277	−0.463	−0.091	0.004	Negative (inverse)
	Summary score of “feeling tired/feeling emotionally exhausted/feeling “dry or puffed” at the end of the day/being on the verge of illness during the pandemic”					
	“feeling tired before/”feeling emotionally exhausted before the pandemic”	0.463	0.213	0.789	0.0001	Positive (in tandem)
	Summary score of “feeling tired/feeling emotionally exhausted/feeling “dry or puffed” at the end of the day & being on the verge of illness during the pandemic”					
	Gender	1.862	1.240	2.484	0.0001	Increasing in males
	Age	−0.598	−0.897	−0.298	0.0001	Negative (inverse)
	Number of children	−0.886	−1.630	−0.142	0.020	Negative (inverse)
	Higher degree	−0.450	−0.802	−0.098	0.012	Negative (inverse)
	Summary score of “I don’t have enough energy for family and friends in my leisure time during the pandemic”					
	Gender	0.435	0.255	0.615	0.0001	Increasing in males
	Age	−0.093	−0.180	−0.006	0.036	Negative (inverse)
	Number of children	−0.360	−0.576	−0.146	0.001	Negative (inverse)
	Higher degree	−0.179	−0.281	−0.077	0.001	Negative (inverse)
	Degree (additional to dentistry)	−0.258	−0.498	−0.019	0.034	Negative (inverse)

**Table 3:** Results on emotional exhaustion because of contact with patients (multiple linear regression modeling).

	Emotional Exhaustion Because of Contact with Patients before and during Pandemic					
Predictors		**B**	**95% CI for B**		**P**	**Association**
	Summary score of “Before the pandemic I found it difficult to deal with patients” &” I feel that working with patients reduces my energy regardless of the period of the pandemic we are experiencing” & ”I believe that I give more than I get when I work with patients regardless of the period of the pandemic we are experiencing” & “I am tired of working with patients regardless of the pandemic period”					
	Age	−0.390	−0.646	−0.133	0.003	Negative (inverse)
	Number of children	−0.822	−1.459	−0.185	0.012	Negative (inverse)
	Summary score of “During the pandemic, I find it difficult to deal with patients” & “I wonder at times how much longer I can continue to work with patients”					
	Summary score of “Before the pandemic I found it difficult to deal with patients” & “I feel that working with patients reduces my energy regardless of the period of the pandemic we are experiencing” & “I believe that I give more than I get when I work with patients regardless of the period of the pandemic we are experiencing” &”I am tired of working with patients regardless of the period of the pandemic we are experiencing”	0.629	0.450	0.823	0.0001	Positive (in tandem)
	Summary score of “During the pandemic I find it difficult to deal with patients” &” I wonder at times how much longer I can continue to work with patients”					
	Gender	0.662	0.336	0.988	0.0001	Increasing in males
	Age	−0.513	−0.670	−0.356	0.0001	Negative (inverse)
	Number of children	−0.701	−1.093	−0.310	0.0001	Negative (inverse)
	Higher degree	−0.207	−0.392	−0.023	0.028	Negative (inverse)
	Years of practice	0.408	0.214	0.602	0.0001	Positive (in tandem)

## Data Availability

The data presented in this study are available on request from the corresponding author. The data are not publicly available due to ethical restrictions.
